# Comparison of diet, lactulose, and metronidazole combinations in the control of pre‐surgical clinical signs in dogs with congenital extrahepatic portosystemic shunts

**DOI:** 10.1111/jvim.16464

**Published:** 2022-05-28

**Authors:** Goncalo Serrano, Nausikaa Devriendt, Hilde de Rooster, Dominique Paepe

**Affiliations:** ^1^ Small Animal Department, Faculty of Veterinary Medicine Ghent University Ghent Belgium

**Keywords:** ammonia, hepatic encephalopathy, liver, medical, portosystemic shunt, treatment

## Abstract

**Background:**

Hepatic supportive diet (HSD), lactulose, and antimicrobials are medical treatments for dogs with congenital extrahepatic portosystemic shunts (cEHPSS). The relative contribution of these treatment components is currently unknown.

**Objectives:**

To determine which treatment combinations are most efficacious in pre‐surgical control of clinical signs of cEHPSS in dogs.

**Animals:**

Thirty‐six dogs with untreated cEHPSS.

**Methods:**

Three‐arm randomized clinical trial. At inclusion (T0), dogs were divided into 3 groups: HSD (n = 12), HSD + lactulose (n = 12), or HSD + metronidazole (n = 12) and received the randomized treatment for 4 weeks (T1) followed by combined treatment of HSD + lactulose + metronidazole for 2 weeks or until cEHPSS attenuation (T2). Clinical score as well as fasting ammonia (FA) and C‐reactive protein (CRP) concentrations were compared among groups and time points.

**Results:**

Thirty‐four dogs were evaluated. Thirty‐four dogs reached T1 and 29 dogs T2. At T1, clinical scores decreased in the HSD + lactulose (n = 11; *P* = .001), but not in the HSD (n = 8; *P* = .96) and HSD + metronidazole (n = 10; *P* = .06) groups. Adding metronidazole to HSD + lactulose (n = 11) did not result in further clinical score improvement (T2; *P* = 1.000). Moderate and weak correlation between clinical score and FA and clinical score and CRP was present (*ρ* = .35, *P* < .001; *ρ* = .27, *P* = .01, respectively) with FA decreasing over time on medical treatment (*P* = .001).

**Conclusions and Clinical Importance:**

Combined HSD + lactulose seems sufficient for pre‐surgical cEHPSS stabilization unlike sole HSD or HSD + metronidazole. Medical treatment of cEHPSS clinical signs decreases FA.

**Abbreviations**
cEHPSS: congenital extrahepatic portosystemic shunt
cPSS: congenital portosystemic shunt
CRP: C‐reactive protein
FA: fasting ammonia
HE: hepatic encephalopathy
HSD: hepatic supportive diet
TSPS: transsplenic portal scintigraphy

## 1 INTRODUCTION

Congenital portosystemic shunts (cPSS) are abnormal vascular communications between the portal and systemic circulation.^1^ This communication allows for toxin‐rich blood to bypass the liver, entering the systemic circulation directly and causing a myriad of clinical signs. Hepatic encephalopathy (HE) is usually the most common clinical sign at presentation, although gastrointestinal and urinary tract signs are also frequently reported.^2^ Several factors are implicated in the HE pathogenesis with ammonia playing a central role, whereas inflammation has been suggested as an important contributing factor.^3^, ^4^ Furthermore, after surgical attenuation of cPSS, C‐reactive protein (CRP) concentration was observed to decrease significantly.^5^

Medical treatment, consisting of a combination of hepatic supportive diet (HSD), lactulose, antimicrobial drugs, or some combination of these, and attenuation by surgery or interventional radiologic procedure have been described as valid treatments for cPSS.^6^ Long‐term survival is reported to be superior in dogs that undergo surgery compared to dogs treated medically.^7^ Notwithstanding its inferiority as long‐term management, medical treatment is an important step in the pre‐surgical stabilization of surgical candidates and in the post‐surgical period when gradual occlusion techniques are used and the cPSS is still patent. This preoperative and postoperative medical management is thought to lead to a reduction of clinical signs, anesthetic risk and perioperative complications.^8^ As mentioned above, medical management is a combination of different treatments, each with a different mechanism of action. Lactulose, a non‐absorbable disaccharide, decreases ammonia production and absorption in the colon by decreasing colonic pH, causing a shift from ammonia to ammonium, with the latter being poorly absorbed by the gastrointestinal mucosa. It also decreases gastrointestinal transit time and inhibits glutaminase activity, decreasing intestinal uptake of glutamine and its metabolism to ammonia.^9^ Antimicrobials such as metronidazole and ampicillin, which have been widely used in veterinary medicine, have their main therapeutic effect in controlling cPSS clinical signs by eliminating urease‐producing bacteria, subsequently decreasing ammonia production and absorption.^10^, ^11^ Hepatic supportive diets, another component of medical management, are traditionally lower in protein, while incorporating highly digestive protein, leaving less protein substrate to be metabolized to ammonia by gastrointestinal bacteria.^12^

Medically treated dogs with cPSS receiving HSD vs HSD and lactulose had no difference in median survival time when followed for 3.2 years.^13^ Additionally, a meta‐analysis in people with HE found that rifaximin, a poorly absorbed antimicrobial, is at least as effective as nonabsorbable disaccharides in controlling clinical signs of HE.^11^ These findings in people and dogs prompt the question of which part of medical management is effective for control of clinical signs in cPSS dogs. Little information is available in the veterinary literature to compare efficacy of different medical treatment components.^6^

To investigate which components of medical management are more effective in pre‐surgical stabilization, a 3‐arm randomized clinical trial was designed, including dogs recently diagnosed with a congenital extrahepatic portosystemic shunt (cEHPSS) that were not receiving any medication or a decreased protein diet. We aimed to compare the effect of HSD, HSD + lactulose and HSD + metronidazole on clinical signs as well as fasting ammonia (FA) and CRP concentrations. A secondary aim was to investigate whether combination treatment (HSD + lactulose + metronidazole) would have superior effects to these 3 treatments on the same variables.

## 2 MATERIAL AND METHODS

### 2.1 Animals

The study was approved by the local ethical and deontological committee (EC2017/49 and DC2017N06), and written consent was obtained from each dog owner before study inclusion. In total, 36 client‐owned dogs with cEHPSS referred to Small Animal Hospital—Ghent University were enrolled. The dogs had a definitive diagnosis of cEHPSS, confirmed either by abdominal doppler ultrasonography, transsplenic portal scintigraphy (TSPS), computed tomography angiography, or some combination of these procedures. Dogs were not included in the study if they had received antimicrobial drugs, lactulose, HSD or probiotics in the 5 days before study inclusion or if they had clinically relevant concurrent disease potentially contributing to the clinical score.

### 2.2 Study design

A 3‐arm, prospective, randomized clinical trial was designed. Dogs were randomly assigned by means of sealed envelopes to 1 of 3 treatments: HSD (Royal Canin hepatic, daily portion divided into 4‐5 meals), HSD + lactulose (Lactulose EG, Eurogenics, Brussels, Belgium; 0.5 mL/kg PO q8h), or HSD + metronidazole (Stomorgyl, Merial, Toulouse, France; 7.5‐10 mg/kg PO q12h). The assigned treatment was advised to be given for 4 weeks (time point 1, T1). After 4 weeks, all dog owners were advised to give a combination of HSD + lactulose + metronidazole for 2 additional weeks or until surgical cEHPSS attenuation (time point 2, T2). A questionnaire was completed by the owner at study inclusion (time point 0, T0), T1 and T2. Dogs with severe uncontrolled clinical signs between T0 and T1 had the combined treatment started earlier than 4 weeks. Dogs experiencing adverse effects attributed to medication started at T0 or T1 had the component suspected of causing such effects decreased in dosage or stopped. Dogs in which drug treatment needed to be changed earlier than the foreseen time points or died of causes attributed to the cEHPSS were given the maximum possible clinical score. Clinical score was included in the next time point (T1 or T2) for statistical purposes. Comparison of clinical scores among groups was performed twice, first including all dogs that completed the clinical trial and again after excluding the dogs that had their allocated treatment modality stopped earlier than the expected timepoints or that died of causes attributed to the cEHPSS. Because no studies comparing different medical treatments for cPSS have been published, a sample size calculation to determine the number of dogs needed to be included in each group could not be performed. A randomly determined number of 12 dogs to be included in each group was set, totaling 36 dogs. These 36 dogs corresponded to the number of dogs with cEHPSS expected to be seen in a 2‐year timespan at our veterinary hospital.

### 2.3 Clinical score

Clinical score was calculated from standardized questionnaires that were filled out by the owners at the different time points (T0, T1, T2), as published before (Table 1).^14^, ^15^ Briefly, any clinical sign occurring often was allocated 2 points and those occurring occasionally were allocated 1 point. The points for gastrointestinal and urinary signs were not multiplied, but those for seizure and coma were multiplied by 3 and all other neurologic signs multiplied by 2, resulting in a maximal clinical sign score of 62. The higher the score, the worse the clinical status.

**TABLE 1:** Clinical scoring system in dogs with an extrahepatic portosystemic shunt^14^, ^15^

	Frequency	Multiplication factor	Maximal score
	often—occasionally—never		
Gastrointestinal signs			
*Salivation, decreased appetite, weight loss, diarrhea, vomiting, melena, hematochezia*	2—1—0 for each gastrointestinal sign	×1 for each gastrointestinal sign	14
Urinary signs			
*Hematuria, stranguria, dysuria, pain while urinating*	2—1—0 for each urinary sign	×1 for each urinary sign	8
Neurological signs			
*Lethargic, unresponsive, disorientation, circling head pressing, ataxia, blindness*	2—1—0 for each neurological sign	×2 for each neurological sign	40
*Seizure activity, comatose*		×3 for each neurological sign	

### 2.4 Blood sampling

Venous blood samples were collected at T0, T1, and T2 for FA and CRP measurements. Venous FA concentration was immediately determined using a commercially available and validated hand‐held ammonia analyzer (PocketChem BA, A. Menarini Diagnostics; upper limit, 45 μmol/L; detection range, 8‐285 μmol/L).^16^ Once blood in the serum tubes was clotted, samples were centrifuged at 3500*g* for 5 minutes and serum was immediately stored at −80°C for later CRP measurement. C‐reactive protein concentrations were measured in batch using a validated commercial kit (Gentian canine CRP reagent kit; upper limit, 10 mg/L; detection range, 10‐1000 mg/L).^17^

### 2.5 Statistical analysis

Statistical analyses were performed using SPSS Statistics 26 (IBM, Armonk). Kruskal‐Wallis tests were performed to assess differences in age, body weight, times receiving the different treatments, clinical score, FA and CRP concentrations, and number of dogs with FA concentrations within reference interval at T0 vs T2. In case of significant differences, Bonferroni corrections were used to take multiple comparisons into account. To assess differences in groups' clinical scores over time, Friedman's two‐way analysis was performed with Bonferroni corrections for multiple comparisons in case of significant differences. A Wilcoxon signed rank test was performed to assess the difference of FA and CRP concentrations at diagnosis vs T2. Subsequently, a Mann‐Whitney *U*‐test was used to assess FA and CRP concentrations in dogs receiving combined treatment during <15 days vs those that received treatment for >25 days. Finally, Spearman correlation tests were used to assess correlations between clinical score on the one hand, and FA and CRP on the other hand, and between FA and CRP. Results were considered significant if *P* ≤ .05.

## 3 RESULTS

### 3.1 Study population at baseline (time point T0)

Between October 2017 and March 2020, 36 dogs were enrolled. Twelve dogs were originally included at T0 in the HSD group, 12 in the HSD + lactulose diet group and 12 in the HSD + metronidazole group. Between T0 and T1, 2 dogs had to be excluded, 1 in the HSD + metronidazole group and 1 in the HSD + lactulose group. The former was euthanized related to paralysis caused by a subarachnoid cyst and the latter was excluded because of uncontrollable central nervous signs present at study inclusion and that persisted almost 3 years after cEHPSS closure was confirmed by imaging. The remaining 34 dogs completed the trial and were used for statistical analysis. Breeds were Maltese (5), Yorkshire terrier (6), Pug (3), Cross breeds (3), Dachshund (3), Boomer (2), Jack Russell terrier (2), Papillon (2), West highland white terrier (2), and a single dog for each of the following breeds: Border collie, Brussels griffon, Keeshond, Miniature pinscher, Shih Tzu, small Münsterländer. Thirteen dogs were intact males and 3 castrated, and 12 were entire female and 6 spayed.

Median age and body weight at T0 are presented in Table 2. No differences in median age or weight were present at T0 (*P* = .36 and .17, respectively).

**TABLE 2:** Median age (range) in days and median weight (range) in kilograms for each treatment group at T0

	HSD (n = 12)	HSD + lactulose (n = 11)	HSD + metronidazole (n = 11)
Age	531.5 (105‐3218)	171 (105‐1460)	340 (82‐1349)
Body weight	2.9 (1.5‐10.2)	2.4 (1‐5.6)	3.4 (1.3‐29.5)

Median clinical scores for each of the groups at T0 are presented in Table 3. At T0, no differences in clinical signs score were present among groups (*P* = .8).

**TABLE 3:** Median (range) clinical score for the different treatment groups at the 3 different time points

	HSD	HSD + lactulose	HSD + metronidazole
T0	15 (8‐36); n = 12	20 (5‐32); n = 11	13 (4‐32); n = 11
T1	7.5 (0‐62); n = 12	1 (0‐20); n = 11	5 (0‐62); n = 11
T2	2.5 (0‐6); n = 8	3 (0‐62); n = 11	4 (0‐22); n = 10

*Note*: Maximum score 62.

Abbreviations: T0, time point 0; T1, time point 1; T2, time point 2.

### 3.2 End of initial treatment (time point T1)

Dogs presented at T1 a median of 29 days (range, 3‐54) after T0. Two dogs in group HSD and 1 in group HSD + metronidazole had to be started earlier on combined treatment because of unsatisfactory control of clinical signs (3, 15, and 8 days after T0, respectively). Another dog in the HSD + metronidazole group had to have the metronidazole discontinued 15 days after study inclusion because of development of tremors when metronidazole was administered. Additionally, a dog in HSD group died the morning before presentation for T1 reevaluation (25 days after T0) after developing severe neurologic signs with the cause of death likely being secondary to HE. All of these 5 dogs were considered treatment failures. In the HSD group, 9 dogs were evaluated between 25 and 34 days and 1 dog after 54 days, the other 2 were described above. One dog was presented 54 days after T0 because the owner considered the dog to be well‐controlled and postponed the evaluation date. In the HSD + lactulose group, all dogs (n = 11) were presented between 25 and 31 days after T0. In the HSD + metronidazole group (n = 11), all dogs were presented 28‐34 days after T0, except the previously mentioned 2. No difference in duration from T0 until T1 was present among groups (*P* = .74). The median clinical scores for the different groups at T1 are presented in Table 3. No differences in median clinical score among groups were present at T1, either when all dogs were included (*P* = .07) or only the dogs not considered treatment failures (*P* = .24) were included in the statistical analysis.

### 3.3 End of the combination treatment (time point T2)

Twenty‐nine dogs reached T2, a median of 18 days (range, 3‐46) after T1. For various reasons, 5 dogs were not included in T2: 1 dog was excluded because a non‐steroidal anti‐inflammatory drug and amoxicillin‐clavulanic were prescribed by the referring veterinarian for a digit swelling (HSD group); a previously mentioned dog died the morning before T1 presentation; for 1 dog the owner was not able to administer tablets, meaning metronidazole could not be given (HSD group); 1 dog that had developed an idiosyncratic reaction to metronidazole administration 15 days after T1 thereafter was not restarted on combined metronidazole + lactulose treatment (HSD + metronidazole group); and in the last excluded dog, the owner was satisfied with the clinical control obtained solely with the HSD and refused additional treatment (HSD group). In total, 8 dogs were included in the HSD group, 11 in HSD + lactulose and 10 in HSD + metronidazole group. One dog in HSD + lactulose group had to have the added metronidazole stopped because of continuous lethargy and vomiting 3 days after its start. The dog recovered after metronidazole was discontinued. This dog was considered a treatment failure. In the HSD group (n = 8), dogs were reevaluated 12‐30 days after T1. In the HSD + lactulose group (n = 11), 9 dogs were reevaluated 12‐33 days after T1, with 2 dogs being evaluated 3 and 6 days after T1. In the HSD + metronidazole group (n = 10), 8 dogs were evaluated 13‐32 days after T1, whereas 2 dogs were reevaluated 36 and 46 days after T1. This longer period of time from T1 to T2 was caused by surgical planning, because T2 corresponded to surgery date in most of the dogs included in this study. Nevertheless, no difference of follow‐up time at T2 was found among groups (*P* = .51).

The median clinical scores for each of the groups at T2 are presented in Table 3. No differences in median clinical score among groups were present at T2, either when all dogs (*P* = .74) or only the dogs not considered treatment failures (*P* = .97) were included in the statistical analysis.

### 3.4 Influence of medical treatments on clinical score over time points

The HSD group had a clinical score decrease at T2 compared to T0 both when all dogs were included (*P* = .01) and when treatment failure dogs were excluded (*P* = .01). The HSD group did not have clinical score changes between T0 and T1 nor between T2 and T1. The combined HSD + lactulose group had a significant decrease in clinical score at T1 compared to T0 either when all dogs were included (*P* = .01) or when dogs considered treatment failures were excluded (*P* = .002). The combined HSD + lactulose group also had a significant decrease in clinical score at T2 when compared to T0 both when all dogs were included (*P* = .01) and when treatment failure dogs were excluded (*P* = .004). No clinical score changes were present between T2 and T1 in the HSD + lactulose group. The combined HSD + metronidazole group had a significant decrease in clinical score at T1 compared to T0 only when dogs considered to be treatment failures were excluded (*P* = .01), but not when all dogs were included. Clinical score decreased at T2 when compared to T0 both when all dogs were included (*P* = .01) and when treatment failures were excluded (*P* = .002) in the HSD + metronidazole group. No changes in clinical score between T2 and T1 were observed in the HSD + metronidazole group.

**FIGURE 1:**
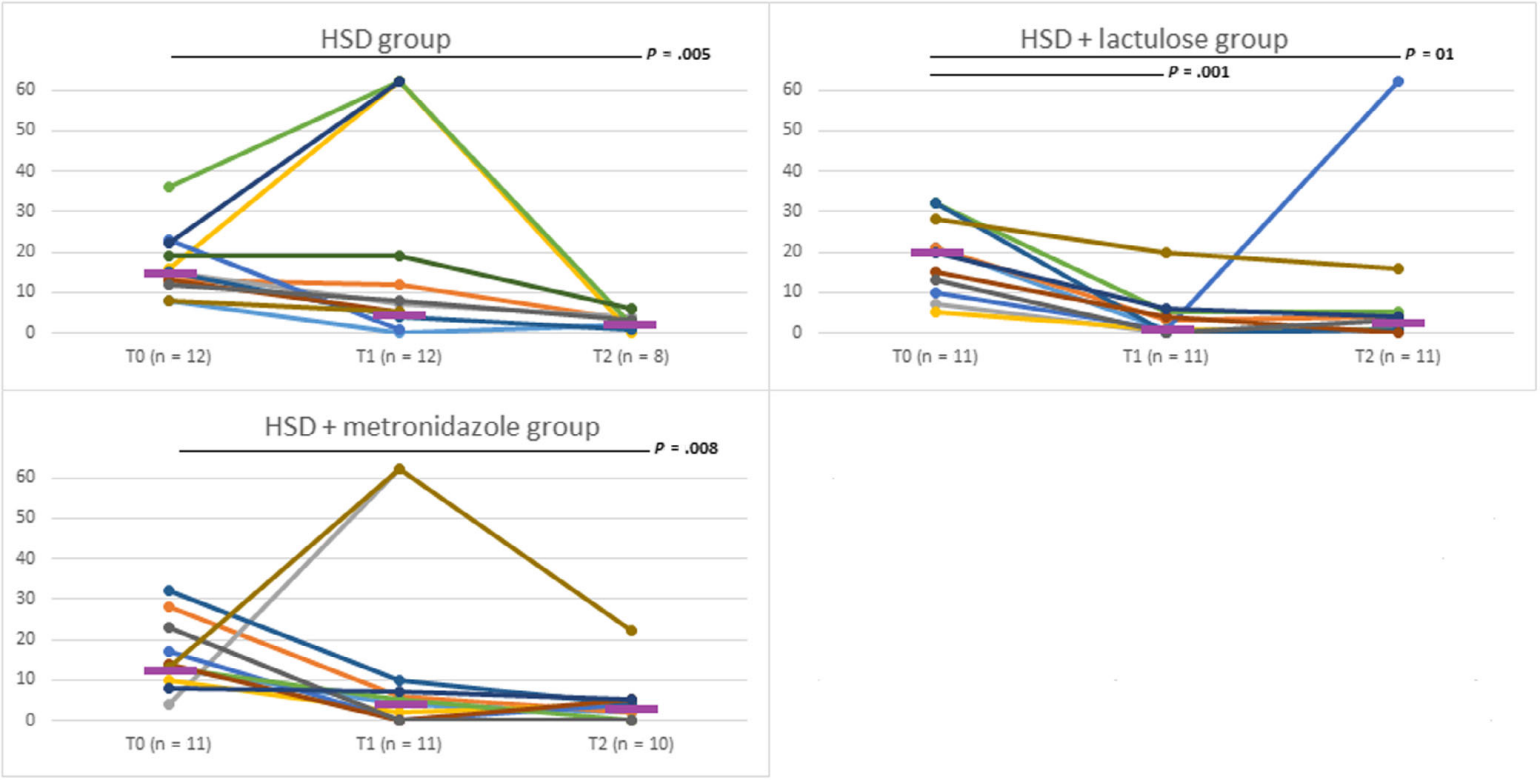
Change in overall clinical score at diagnosis (T0), time point 1 (T1), and time point 2 (T2) for all included dogs. Each line represents one dog. The purple transverse line in the figure represents the median score. Statistically significant changes in overall clinical score are shown by the black line over the figure

Statistical comparisons between different time points are presented in Table 4. Figure 1 shows the changes in clinical score when all dogs were included.

**TABLE 4:** Statistical differences (*P*‐values) in clinical scores over time for the different treatment groups

	HSD		HSD + lactulose		HSD + metronidazole	
	All dogs (n = 8)	Failures excluded (n = 6)	All dogs (n = 11)	Failures excluded (n = 10)	All dogs (n = 10)	Failures excluded (n = 9)
T0‐T1	.96	.25	.001	.002	.06	.01
T1‐T2	.1	.58	1.00	1.00	1.00	1.00
T0‐T2	.01	.01	.01	.004	.01	.002

*Note*: Statistical differences are shaded in gray.

Abbreviations: T0, time point 0; T1, time point 1; T2, time point 2.

### 3.5 Fasting ammonia and C‐reactive protein concentration

At study inclusion, FA concentration results were available in 32 dogs with 4 having FA within the reference interval. At T1, FA concentration was available in 29 dogs (27 dogs that completed the clinical trial and 2 dogs considered treatment failures) with 4 having FA within the reference interval. Fasting ammonia concentration at T1 was measured a median of 29 days (range, 14‐54) after T0, in all except 2 dogs, in which it was measured between 25 and 34 days. At T2, FA concentrations were available in 28 dogs (26 dogs that completed the clinical trial and 2 dogs considered treatment failures) with 8 being within the reference interval. When including all dogs, FA at T2 was measured a median of 18 days (range, 6‐46) after T1. In 8 dogs, it was measured between 12 and 15 days, between 16 and 19 days in 7, 22 and 25 days in 5, and 29 and 36 days in 6 dogs. Fasting ammonia concentrations for the 3 groups at the different time points are presented in Table 5.

**TABLE 5:** Median (range) fasting venous ammonia concentration (μmol/L) for the different treatment groups at the 3 different time points

	HSD	HSD + lactulose	HSD + metronidazole
T0	133 (17‐high); n = 11	173 (66‐high); n = 11	107.5 (43‐high); n = 11
T1	142 (22‐196); n = 9	136 (36‐high); n = 10	117 (21‐199); n = 9
T2	75 (19‐146); n = 8	107 (30‐256); n = 10	73.5 (21‐176); n = 10

Median FA concentration did not differ among groups at T0, T1, or T2 (*P* = .44, .58, or .4, respectively, with all dogs included and *P* = .44, .66, or .16, respectively, with failed dogs excluded). No significant difference was found between the number of dogs with FA concentrations within the reference interval at T0 compared to T2 (*P* = .21), but FA concentrations decreased significantly at T2 when compared to T0 (*P* = .001). A moderate positive correlation between FA and clinical score was present both when all dogs were included and when the treatment failure dog was excluded (*P* < .001 and *ρ* = .35 and *P* = .003 and *ρ* = .31, respectively). To assess if the duration of combined treatment was the cause for the decreased FA at T2 when compared to T1, dogs that had ≤15 days (n = 9; median time to T2 = 13 days; median FA = 66 μmol/L) from T1 to T2 were compared to dogs that had a T1 to T2 time >25 days (n = 9; median time to T2 = 30 days; median FA = 65 μmol/L). Median FA concentration was not significantly different between dogs receiving the combined treatment for a shorter (maximum, 15 days) vs longer period (minimum, 25 days; *P* = .67).

Serum for CRP quantification was frozen at −80°C for a median of 27 months (range, 12‐45 months). Median CRP concentrations for the 3 groups at the different time points can be found in Table 6. At T0, serum CRP concentration was measured in 33 dogs with 28 dogs having a within reference interval CRP concentration (<10 mg/L). At T1, serum CRP concentration was measured in 29 dogs (including a dog considered a treatment failure) with 26 dogs having a within reference interval CRP concentration. At T2, serum CRP concentration was measured in 25 dogs (including 3 dogs considered treatment failures) with all but 1 dog having a within reference interval CRP concentration.

**TABLE 6:** Median (range) CRP concentration (mg/L) for the different treatment groups at the 3 different time points

	HSD	HSD + lactulose	HSD + metronidazole
T0	<10 (<10‐10.1); n = 12	<10 (<10‐42.5); n = 11	<10 (<10‐13.8); n = 10
T1	<10 (<10‐58.9); n = 9	<10 (<10‐30.2); n = 11	<10 (<10); n = 9
T2	<10 (<10); n = 9	<10 (<10‐13.6); n = 7	<10 (<10); n = 9

No differences in median CRP concentration among groups were present at T0, T1, or T2 (*P* = .36, .45, or .28, respectively, with all dogs included and *P* = .36, .45, or .34, respectively, with failed dogs excluded). No change in CRP concentration at T2 compared to T0 was present (*P* = .66). A positive, weak correlation between serum CRP concentration and clinical score was present both when all dogs were included and when the treatment failure dogs were excluded (*P* = .01 and *ρ* = .27; *P* = .01 and *ρ* = .27, respectively). Similar to FA, in order to assess if combined treatment would affect CRP concentration, dogs that had ≤15 (n = 7; median time to T2 = 14 days; median CRP concentration < 10 mg/L) from T1 to T2 were compared to dogs that had a T1 to T2 > 25 days (n = 8, median time to T2 = 30 days; median CRP concentration < 10 mg/L). Median CRP concentration was not significantly different between dogs receiving the combined treatment for a shorter (maximum, 15 days) vs longer period (minimum, 25 days; *P* = 1.00).

No correlation between FA and CRP concentration was present (*P* = .14).

## 4 DISCUSSION

Our prospective, randomized clinical trial assessing different medical treatment modalities in cEHPSS dogs had several interesting findings: (a) HSD + lactulose was effective in the pre‐surgical control of clinical score; (b) adding metronidazole to HSD + lactulose did not result in further improvement of clinical score; and (c) improvement of clinical score is associated with a decrease in FA concentrations.

The absence in a significant decrease in clinical score from T0 to T1 suggests that HSD alone does not seem to be sufficient to control clinical signs in many dogs. This finding suggests that the protein reduction typical with such a diet alone might not always have enough impact on intestinal production of ammonia or other toxic components in dogs with cEHPSS. This finding is in opposition to what was described in literature, in which improvement of clinical signs was seen when HSD alone was used.^12^, ^13^ We cannot completely rule out that the inferiority of HSD in controlling the clinical signs might be related to the way food was provided. Although owners were advised to divide the daily portion (calculated for the dog's body weight) in 4 to 5 meals,^18^ it is not possible to know how rigorously these instructions were followed. Nevertheless, the way food is administered by owners represents the reality of cEHPSS stabilization, making the observed results regarding HSD use applicable and appropriate to the general cEHPSS population. In addition, the time needed for HSD to start controlling clinical signs also could be a reason for the lower efficacy of HSD vs HSD + lactulose, but in our observations from this and previous studies, dogs that showed little to no response to the HSD a few days after its start did not seem to have a satisfactory response after several weeks. Nevertheless, it was not our objective to assess how many days of treatment are needed to achieve clinical normalization with the different medical treatments, and additional studies are needed to answer this question. Another limitation is that we only evaluated a single type of diet, and perhaps other commercial or home‐cooked diets along with the consultation of a veterinary nutritionist would be more effective in controlling clinical signs in dogs with cEHPSS.^12^ Adding lactulose to HSD might have additive effects when compared to lactulose administration alone, by acidifying the intestinal lumen and decreasing the gastrointestinal transit time while the protein content that reaches the colon is decreased. Lactulose efficacy when administered without protein‐restricted food might be lower than when administered with protein‐restricted food. Therefore, HSD still is advised as a standard food in combination with lactulose.

An important finding was that adding metronidazole to HSD + lactulose did not further decrease the clinical signs. These observations support the use of HSD + lactulose as a sufficient pre‐surgical stabilizing treatment protocol for cEHPSS. We chose to use metronidazole as a antimicrobial drug because it is widely used in veterinary clinical practice and has been recommended as a therapeutic component in the medical management of cPSS.^11^ In healthy dogs, metronidazole has excellent bioavailability, being distributed to most tissues and body fluids after PO administration.^19^ Of note, metronidazole has not been advocated in the treatment of HE in people because of its prolonged elimination in the presence of hepatic dysfunction and increased risk of irreversible peripheral neuropathies.^20^, ^21^ In our study, 1 dog developed neurologic signs (tremors) when metronidazole was started and another developed lethargy and vomiting that resolved when metronidazole was discontinued. Both dogs were siblings, making an idiosyncratic reaction possible.

We conclude that metronidazole did not have additional benefits over lactulose. Nevertheless, other antimicrobials, such as rifaximin, might have added value in cPSS stabilization. Rifaximin has been shown to be as effective as metronidazole in the management of acute overt type C HE (HE resulting from chronic liver disease) in people.^22^ Furthermore, a recent meta‐analysis in people with type C HE identified a clinical benefit when rifaximin and lactulose were combined compared with lactulose in decreasing mortality and clinical signs.^23^ Type C HE has a different underlying pathology than PSS‐related HE and conclusions regarding antimicrobial use might therefore not translate to type B HE (the type of HE in cPSS, in which no underlying liver parenchymal disease is present). Although it would be interesting and pertinent to include a group receiving rifaximin, its current price precludes routine use in veterinary medicine. Furthermore, from the data obtained in our study, HSD + lactulose without the addition of metronidazole seems to be an acceptable treatment protocol for cEHPSS dogs, avoiding the potential adverse effects and additional costs associated with metronidazole. More importantly, decreased antimicrobial usage contributes to decreased overall antimicrobial resistance of bacteria in animals as well as people.^24^

From the 11 dogs receiving HSD + lactulose that reached T1, all but 1 had noticeably decreased clinical signs, and adding metronidazole to the initial treatment protocol of the only dog described as having a higher clinical score at T1 also failed to improve its clinical score (Figure 1).

Medical treatment for cPSS was shown to decrease the severity of clinical signs, as has been described before,^12^ and to decrease FA concentration which, to our knowledge, has not been reported previously in dogs. Additionally, the number of dogs with FA concentrations within the reference interval increased from T0 to T2. This difference however was not significant, probably because of the small number of dogs included (type 2 error). This finding may be important in clinical practice, because starting medical treatment before confirmation of cPSS might hamper the use of FA as a screening test for cPSS.

The previously described association of CRP^4^ with clinical signs also was observed in our study, with median CRP concentration being weakly correlated with clinical score. Despite the weak correlation found, most of the dogs had CRP concentrations lower than the reference interval, and lower than the detection limit of the assay. We can hypothesize that if the reference interval of the currently used CRP assay had been lower, the results could had been different. Additional studies with a larger population and an assay with a lower reference interval potentially would allow confirmation of the positive correlation between clinical signs and CRP in dogs treated medically, as has been described in dogs undergoing surgical cPSS attenuation.^5^ In people, CRP is stable for up to 34 months in frozen serum,^25^ making it unlikely that the present CRP concentrations were erroneously low because of overly long storage.

The duration of medical treatment could be the main reason for the decrease in FA. Therefore, the FA concentrations of dogs with shorter (maximum, 15 days) vs longer (minimum, 25 days) duration between T1 to T2 were compared and found not to be significantly different. These findings were based on a small population, which makes type 1 error possible. Another explanation would be the synergistic effect of the combined treatment in decreasing the FA concentration. Additional studies with a larger population of dogs followed for longer periods of time are necessary to determine whether duration of treatment or the combined treatment effect is the reason for the decrease in FA concentrations.

Our study focused on pre‐surgical cPSS stabilization, which should be differentiated from long‐term cPSS management. Most of the dogs included in our study were followed for <2 months and it is important not to overinterpret the data and conclude that antimicrobials also could not have additional benefit over the combination HSD + lactulose for the long‐term medical management of cPSS. Additional studies regarding the usefulness of antimicrobial treatment as a component of the long‐term medical management of cPSS are needed.

Our study had some limitations. The population included was small, which could lead to type 1 or type 2 errors. A sample size calculation was not possible initially because of the absence of data comparing different medical treatment components. Nevertheless, our results will facilitate sample size calculations in future studies aiming to compare different medical treatment components. Second, the clinical score was assessed by the owner, which might introduce bias. Clinical scores given might be influenced by owners' expectations or interpretations of their dogs' behavior, which are subjective and may not always be realistic. Furthermore, owners and the investigators were not blinded to the treatment given. This factor could affect interpretation of the efficacy of different medical treatment components based pre‐conceived beliefs of the owners or investigators regarding the efficacy of different medical treatment components. Predefined guidelines of what to consider a treatment failure and how to manage adverse events could have decreased owner or clinician bias or both. Third, in some dogs, time to T1 was shorter and combined treatment was started earlier. These shorter times to T1 were a result of uncontrollable clinical signs or dogs not tolerating the prescribed medication. Because the dogs were privately owned, the starting or stopping of medications involves discussion with the owner and is influenced by owner's willingness to continue the treatment the dog was allocated despite persistent clinical signs. Different owners might have different definitions of what is tolerable or not and might wish to start combined treatment earlier or might be content to wait until T1 is completed. Nevertheless, all dogs that reached T1 earlier than expected did so because of uncontrollable clinical signs that were not expected to subside, even with continuation of the allocated treatment for a longer period. These dogs were considered treatment failures and, in order to represent treatment failure, a “worst observation carried forward” approach was chosen, meaning that a maximum possible score was assigned for the failed time point. We also presented the data without including the dogs considered treatment failures because doing so allows clinicians to assess and decide which treatment combinations are safest. The exclusion of treatment failures from statistical analysis however induces a significant bias, and this data that excludes treatment failures must be interpreted carefully. Fourth, T2 was variable in the dogs included, although no difference in follow‐up time among groups was detected. If all dogs included at T2 had similar follow‐up time, conclusions regarding the effect of combined treatment would be stronger. We tried to standardized T2 time, but because of the dogs being privately owned and as a result of clinical practice organization (eg, availability of surgery) a wider dispersion of time at T2 occurred. Fifth, the 5‐day period without having received HSD, lactulose, antimicrobials, or probiotics before study inclusion might have been too short. Recent data indicate that antimicrobials might alter the gastrointestinal microbiota several weeks after discontinuation.^26^ This finding means that the amount of ammonia produced by intestinal bacteria and associated clinical signs of HE may be lower several weeks after antimicrobial discontinuation. Ideally, all included dogs should not have received any medications before referral, but doing so would limit study size too much. Although the majority of dogs presented had high clinical scores at T0, decreases in clinical score before and after treatment might have been underestimated by this study limitation.

In conclusion, the combination of HSD with lactulose provided appropriate control of clinical signs in the studied population and it seems reasonable to suggest that metronidazole can be omitted from the treatment protocol for dogs with cEHPSS awaiting surgical cEHPSS attenuation. However, further work is needed to allow more specific conclusions about the preferred pre‐surgical medical management of dogs with cEHPSS.

## CONFLICT OF INTEREST DECLARATION

Authors declare no conflict of interest.

## OFF‐LABEL ANTIMICROBIAL DECLARATION

Metronidazole used off‐label.

## INSTITUTIONAL ANIMAL CARE AND USE COMMITTEE (IACUC) OR OTHER APPROVAL DECLARATION

Approved by Ghent University, EC2017/49 from 7th September, 2017 and DC2017N06 from 7th November, 2017.

## HUMAN ETHICS APPROVAL DECLARATION

Authors declare human ethics approval was not needed for this study.
